# Meiotic Recombination in Human Oocytes

**DOI:** 10.1371/journal.pgen.1000661

**Published:** 2009-09-18

**Authors:** Edith Y. Cheng, Patricia A. Hunt, Theresa A. Naluai-Cecchini, Corrine L. Fligner, Victor Y. Fujimoto, Tanya L. Pasternack, Jackie M. Schwartz, Jody E. Steinauer, Tracey J. Woodruff, Sheila M. Cherry, Terah A. Hansen, Rhea U. Vallente, Karl W. Broman, Terry J. Hassold

**Affiliations:** 1 Department of Obstetrics and Gynecology, University of Washington, Seattle, Washington, United States of America; 2 School of Molecular Biosciences, Washington State University, Pullman, Washington, United States of America; 3 Department of Pathology, University of Washington, Seattle, Washington, United States of America; 4 Department of Obstetrics and Gynecology and Reproductive Sciences, University of California San Francisco, San Francisco, California, United States of America; 5 Department of Biostatistics and Medical Informatics, University of Wisconsin–Madison, Madison, Wisconsin, United States of America; Stowers Institute for Medical Research, United States of America

## Abstract

Studies of human trisomies indicate a remarkable relationship between abnormal meiotic recombination and subsequent nondisjunction at maternal meiosis I or II. Specifically, failure to recombine or recombination events located either too near to or too far from the centromere have been linked to the origin of human trisomies. It should be possible to identify these abnormal crossover configurations by using immunofluorescence methodology to directly examine the meiotic recombination process in the human female. Accordingly, we initiated studies of crossover-associated proteins (e.g., MLH1) in human fetal oocytes to analyze their number and distribution on nondisjunction-prone human chromosomes and, more generally, to characterize genome-wide levels of recombination in the human female. Our analyses indicate that the number of MLH1 foci is lower than predicted from genetic linkage analysis, but its localization pattern conforms to that expected for a crossover-associated protein. In studies of individual chromosomes, our observations provide evidence for the presence of “vulnerable” crossover configurations in the fetal oocyte, consistent with the idea that these are subsequently translated into nondisjunctional events in the adult oocyte.

## Author Summary

One of the defining events in the formation of eggs and sperm is meiotic recombination, a process in which DNA is exchanged between “partner” chromosomes. If this process is perturbed, chromosomes often go astray during meiotic division, resulting in eggs or sperm with too many or too few chromosomes. In humans, the resulting embryos are almost always abnormal and are a major source of miscarriages or congenital birth defects (e.g., Down syndrome). Over the past decade, techniques have become available that make possible the examination of meiotic recombination “as it happens” in the human egg, and this has allowed us to define the properties of this process. In the present study, we used this methodology to characterize meiotic recombination in human fetal oocytes and to ask whether we could identify abnormal recombination patterns. Such patterns were, indeed, observed for each chromosome we studied, although different abnormalities were observed among the individual chromosomes. Thus, factors operating before birth influence the likelihood of chromosome misdivision in the adult oocyte, although those that make chromosome 21 misdivide (leading to Down syndrome) may not be the same as those that make other chromosomes misdivide.

## Introduction

Meiotic errors generate an extraordinary number of chromosome abnormalities in humans, with most of the abnormalities originating in the first meiotic division in the oocyte [1]. Studies conducted over the past 10–20 years have identified the first molecular correlate of these abnormalities, as disturbances in meiotic recombination have been linked to a variety of human trisomies of maternal origin [1]. Specifically, reductions in recombination have been associated with nondisjunction of chromosomes 13, 15, 16, 18, 21, 22 and the sex chromosomes [2],[3],[4],[5],[6],[7],[8],[9],[10], and alterations in the location of crossover events with trisomies 16 and 21 and sex chromosome trisomies [5],[8],[10]. These associations are consistent with similar observations in model organisms such as Drosophila [11] and S. cerevisiae [12], but the magnitude of the effect in humans has been surprising. For example, the majority of cases of trisomy 21 appear to be attributable to failure to crossover, or to crossovers located too close to, or too far away from, the centromere [13]. Similarly, most cases of trisomy 16 are associated with distally located exchanges [5]. Thus, it appears that a large proportion, if not a majority, of human trisomies involve achiasmate homologous chromosomes, or homologous chromosomes with sub-optimally located exchanges.

These observations present a conundrum. That is, how can aberrant meiotic recombination, an event that occurs in the fetal ovary, be a major contributor to trisomy when advancing maternal age, a process that occurs decades later, is unquestionably the most important risk factor for meiotic nondisjunction? The answer is not yet obvious, but several groups have suggested that there is a two-step process that links the two etiological factors [14]. Specifically, it is assumed that a proportion of homologs are either achiasmate or tethered by sub-optimally located crossovers. With increasing maternal age, these configurations become more likely to nondisjoin. If this is the case, at least two predictions follow: First, for older women, chromosome-specific genetic maps constructed from analyses of trisomic conceptions should be shorter, and with a different distribution of exchanges, than those from chromosomally normal offspring. Second, direct analyses of crossover events in human fetal oocytes should reveal “vulnerable” crossover configurations that should exhibit chromosome specificity consistent with data from trisomic conceptions (e.g., for chromosome 16, distally located exchanges and for chromosome 21, proximal and distal exchanges, as well as achiasmate chromosomes).

Over the past several years, a number of groups have attempted to address the first prediction, examining the relationship between crossing-over patterns and maternal age in trisomic conceptions (e.g., [3],[4],[8],[15]). However, there has been little attempt to address the second prediction. In part this is attributable to the obvious challenges in obtaining the appropriate study material (i.e., prophase stage oocytes from fetal ovaries); however, more importantly, there has been no simple approach to visualize the crossing-over process. The introduction of immunofluorescence methodology eliminates this problem and provides a simple, straightforward approach to the analysis of human meiosis, making it possible to monitor the formation of meiosis-specific structures (e.g., the synaptonemal complex, SC) and to visualize interactions between homologs as they pair, synapse and recombine during meiotic prophase. Of particular importance has been the employment of the MutL homologue, MLH1, to identify the sites of crossovers on meiotic chromosomes. Studies of male and female mice and human males indicate a 1∶1 correspondence between the occurrence of MLH1 foci in pachytene spermatocytes/oocytes and sites of chiasmata in diakinesis stage cells or sites of recombination inferred from genetic linkage studies (for review, see [16]). Consequently, by using MLH1 as a surrogate for exchanges, it now seems possible to analyze crossing-over “as it happens” during pachytene. Further, the inclusion of chromosome-specific fluorescence in situ hybridization (FISH) in such investigations provides a means to monitor recombination on individual chromosomes.

Utilizing this approach, several groups have initiated studies of human female meiosis [17],[18],[19],[20] but in most of these the focus has been on the meiotic process, and not specifically on recombination. Thus, we recently initiated studies utilizing human fetal ovarian samples. We evaluated the utility of MLH1 as a marker of crossing-over in females and asked whether achiasmate chromosomes and sub-optimal crossover patterns are, indeed, a feature of human female meiosis. In this report we summarize results on an initial series of 1035 prophase oocytes from 31 fetal ovarian samples. Our results provide evidence of temporal differences between human males and females in the appearance of MLH1 foci on the synaptonemal complex, but indicate that MLH1 foci are a useful marker of crossovers in females as well as males. The results of analyses of the number and location of MLH1 foci provide evidence for the presence of “vulnerable” crossover configurations, observations consistent with data from previous human trisomy studies.

## Results

Preparations of prophase oocytes were made from 31 fetal ovarian samples collected from female fetuses with gestational ages between 14–23 weeks. A summary of the information on these cases is provided in Table 1.

**Table 1:** Summary of patient information from 31 fetal ovarian samples.

ID	Maternal Age (years)	Gestational Age (weeks)	Clinical Observations/Reason for Ascertainment	Chromosome Constitution
EC 010		18		46,XX
EC 018		22		46,XX
EC 041		16	anencephaly	
EC 053		16		
EC 069		20	multiple congenital anomalies	46,XX
EC 076		19	neurotube defect	
EC 091		21		
EC 096		20	renal agenesis with anhydramnios	46,XX
EC 098		17	anencephaly	
EC 099		19		
EC 101		20	maternal vaginal lymphoma	
SF 001	21	20	elective termination	
SF 002	21	21	elective termination	
SF 004	27	20	elective termination	
EC 141		15		
SF 008	25	19	elective termination	
EC 143		14		
SF 009	31	23	elective termination	
EC 147		18	placenta accreta	
SF 010	30	19	elective termination	
SF 011	18	23	elective termination	
SF 012	29	22	elective termination	
SF 013	21	15	elective termination	
SF 018	32	23	elective termination	
SF 020	31	19	elective termination	
SF 023	18	21	elective termination	
SF 024	37	19	elective termination	
SF 025	21	19	elective termination	
SF 029	16	23	elective termination	
SF 032	24	22	elective termination	
SF 035	19	20	elective termination	

### Temporal aspects of MLH1 localization

**Figure 1:**
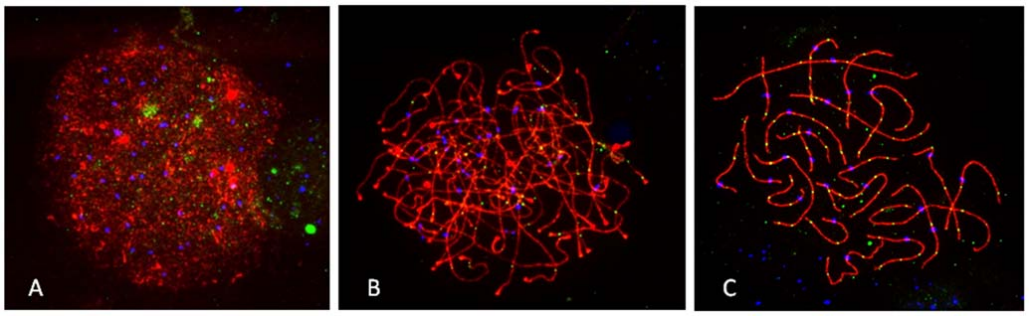
Representative images from (A) leptotene, (B) zygotene, and (C) pachytene stage human fetal oocytes. Antibodies against SYCP3 (representing the axial elements of the synaptonemal complex) are visualized in red and against the DNA mismatch repair protein MLH1 in green, and CREST antiserum-positive signals (recognizing centromeric regions) are visualized in blue.

In previous studies of human males [20],[21],[22], mouse males [23],[24] and mouse females [25], MLH1 has been observed to localize primarily to SCs of pachytene stage cells. However, similar immunostaining studies of human fetal oocytes have suggested a more “relaxed” temporal pattern, with MLH1 foci evident in earlier meiotic stages [17],[26]. Thus, in initial analyses, we were interested in examining the temporal pattern of MLH1 localization in our own series. Representative images of leptotene, zygotene and pachytene cells are provided in Figure 1A–1C. At leptotene (Figure 1A), MLH1 foci were occasionally observed as small, dispersed signals throughout the nucleus, but it was unclear whether these were actually associated with SCs. However, at zygotene (Figure 1B), SC-associated MLH1 foci were clearly visible in all cases that we examined, although the signals were not nearly as intense as those in later pachytene stage cells (Figure 1C). We made no systematic attempt to quantify the number of foci present in zygotene cells, but in general they were less abundant than in pachytene cells.

### Analyses of recombination over the entire genome

Table 2 provides a summary of the mean genome-wide MLH1 counts per cell for the 31 ovarian samples (see Figure S1 for graphical representation of the data). The overall mean MLH1 value per cell for the 1035 pachytene cells we examined was 69.3±14.3. However, there was considerable variation within and among individual ovarian samples. For example, for most ovaries there was an approximate two-fold difference between the lowest and highest MLH1 values. Similarly, there was highly significant variation among the 31 samples (F = 19.0; p<0.0001), with individual mean values ranging from a low of 52.6 (SF 009) to a high of 88.3 (EC 101). This variation was not attributable to either gestational or maternal age, as we saw no obvious relationship between MLH1 counts and these variables. Further, there was no obvious effect of fetal abnormalities (i.e., identified in cases EC 41, 69, 76, 96 and 98; Table 1) on the number or distribution of MLH1 foci.

**Table 2:** Summary of MLH1 analyses from 31 fetal ovarian samples.

ID	Number of Cells	Mean MLH1 Count±S.D.	Range
EC 010	25	59.3±10.0	43–81
EC 018	7	80.7±18.1	53–96
EC 041	71	65.1±11.5	40–89
EC 053	14	69.8±12.4	46–89
EC 069	40	68.2±8.8	44–83
EC 076	36	71.7±10.9	54–95
EC 091	39	68.6±9.4	49–92
EC 096	60	79.6±10.7	59–102
EC 098	30	82.1±10.2	57–107
EC 099	37	87.1±16.0	50–115
EC 101	53	88.3±10.3	66–109
SF 001	59	59.2±14.0	27–89
SF 002	34	59.7±10.4	37–74
SF 004	17	59.1±13.9	40–96
EC 141	13	76.3±14.1	58–94
SF 008	70	66.6±11.6	42–92
EC 143	11	87.2±11.1	64–104
SF 009	13	52.6±10.5	40–61
EC 147	40	67.8±11.0	48–88
SF 010	54	65.5±12.8	45–100
SF 011	10	75.0±8.8	55–89
SF 012	10	64.8±8.0	52–76
SF 013	40	66.4±13.9	43–97
SF 018	12	72.2±14.6	56–107
SF 020	11	60.2±10.8	48–74
SF 023	20	64.3±12.6	45–86
SF 024	39	61.8±10.8	45–84
SF 025	46	59.9±9.7	43–77
SF 029	18	69.6±10.9	55–92
SF 032	50	73.3±11.6	51–103
SF 035	56	69.9±10.3	50–100
**Total**	**1035**	**69.3±14.3**	**27–107**

In a second analysis, we were interested in determining the number of cells in which there were one or more chromosomes without MLH1 foci (assuming a 1∶1 correspondence between MLH1 foci and crossovers, such situations would yield achiasmate chromosomes at metaphase/anaphase I). This assessment was complicated by the length of the individual SCs, which resulted in numerous overlapping SCs in most cells. Nevertheless, we were able to independently visualize all 23 SCs in 176 (17.0%) of the 1035 cells; Figure 1C provides an example of one such cell. In the vast majority (132/176 = 75.0%) of these cells, MLH1 foci were detected on all bivalents. However, in 31 cells we observed a single “MLH1-less” bivalent, in 9 cells two of the SCs were lacking MLH1 foci, in one cell 3 bivalents were without foci and in one cell 6 SCs were lacking foci. We made no attempt to identify the specific MLH1-less chromosomes using FISH; however, on the basis of morphology, exactly one-half involved small acrocentric chromosomes (i.e., either chromosomes 21 or 22), with no other chromosome group being obviously over-represented.

### Chromosome-specific recombination studies

In subsequent studies, we were interested in assessing recombination patterns on chromosomes known to be associated with clinically relevant human trisomies. Thus, we examined chromosomes associated with clinical syndromes (i.e., trisomies 13, 18 and 21), or high frequencies of occurrence in spontaneous abortions (i.e., trisomies 16 and 22); additionally, we included chromosome 17 as a “comparison” for the other E group chromosomes (i.e., chromosomes 16 and 18). Cumulatively, these six chromosomes account for well over 50% of all clinically recognized trisomies [27].

For these analyses, we examined slides from a subset of the study population (i.e., cases EC 69, 76, 91, 96, 98, 99 and 101). These cases were not selected in any way, but instead represented seven consecutive samples collected at the time at which we were interested in conducting this specific analysis. The pooled genome-wide MLH1 mean value for these seven cases was slightly higher than for the general study population (i.e., 77.9 vs. 69.3). However, the data on the individual chromosomes was similar among the seven cases, despite the fact that the mean MLH1 values varied from 68.2 to 88.3; additionally, the proportion of achiasmate chromosomes for these individuals was similar to that observed for individuals in the general series. Thus it seems likely that, in general, the data are representative of the study population as a whole.

#### Chromosome-specific genetic lengths

Table 3 shows the number of MLH1 foci per bivalent and the estimated genetic lengths (assuming that one MLH1 focus = one crossover = 50 cM) for chromosomes 13, 16, 17, 18, 21 and 22. The overall number of MLH1 foci was virtually identical for SCs of chromosomes 13, 16 and 17, with each averaging approximately 2.5 foci per bivalent. The number of foci was slightly reduced for chromosome 18, at 2.2 foci, and chromosomes 21 and 22 each averaged slightly more than one focus, with chromosome 22 somewhat higher than chromosome 21.

**Table 3:** Number of MLH1 foci on individual chromosomes.

Chromosome (number cells)	Number exchanges (%)							Mean/cell	Genetic length (cM)
	0	1	2	3	4	5	6		
13 (104)	1	8	42	39	12	2	–	2.57	128.4
	(1.0)	(7.7)	(40.4)	(37.5)	(11.5)	(1.9)	–		
16 (73)	–	5	30	35	3	–	–	2.49	124.5
	–	(6.8)	(41.1)	(47.9)	(4.1)	–	–		
17 (98)	1	12	31	42	9	2	1	2.57	128.6
	(1.0)	(12.2)	(31.6)	(42.9)	(9.2)	(2.0)	(1.0)		
18 (112)	3	17	53	30	8	1	–	2.23	111.6
	(2.7)	(15.2)	(47.3)	(26.8)	(7.1)	(0.9)	–		
21 (164)	8	115	40	1	–	–	–	1.21	60.4
	(4.9)	(70.1)	(24.4)	(0.6)	–	–	–		
22 (144)	9	87	41	7	–	–	–	1.32	66.0
	(6.3)	(60.4)	(28.5)	(4.9)	–	–	–		

In general, we found that most chromosome arms possessed at least one MLH1 focus (Table 4). The most notable exceptions were the short arms of the acrocentric chromosomes (13, 21 and 22), on which foci were detected in only 11/412 cells (2.7%). Additionally, the short arm of chromosome 18 exhibited a surprisingly high proportion of “MLH1-less” events, as in 42/112 cells (37.5%) either both the p and q arms or the p arm lacked MLH1 foci. This contrasted sharply with the other two E group chromosomes, as for chromosomes 16 and 17 the frequencies of MLH1-less short arms were 0% and 18.3%, respectively.

**Table 4:** Presence or absence of exchanges on arms of individual chromosomes.

Chromosome (no cells)		Presence (+) or absence (−) of MLH1 focus(i) on individual arms of bivalent			
		p arm−/q arm−	p arm+/q arm−	p arm−/q arm+	p arm+/q arm+
13 (104)	no.	1	0	99	4
	(%)	(1.0)	(0.0)	(95.2)	(3.8)
16 (73)	no.	0	5	4	64
	(%)	(0.0)	(6.8)	(5.5)	(87.7)
17 (98)	no.	1	5	17	75
	(%)	(1.0)	(5.1)	(17.3)	(76.5)
18 (112)	no.	3	3	39	67
	(%)	(2.7)	(2.7)	(34.8)	(59.8)
21 (164)	no.	8	2	152	2
	(%)	(4.9)	(1.2)	(92.7)	(1.2)
22 (144)	no.	9	0	132	3
	(%)	(6.3)	(0.0)	(90.3)	(2.1)

#### Chromosomal location of MLH1 foci

**Figure 2:**
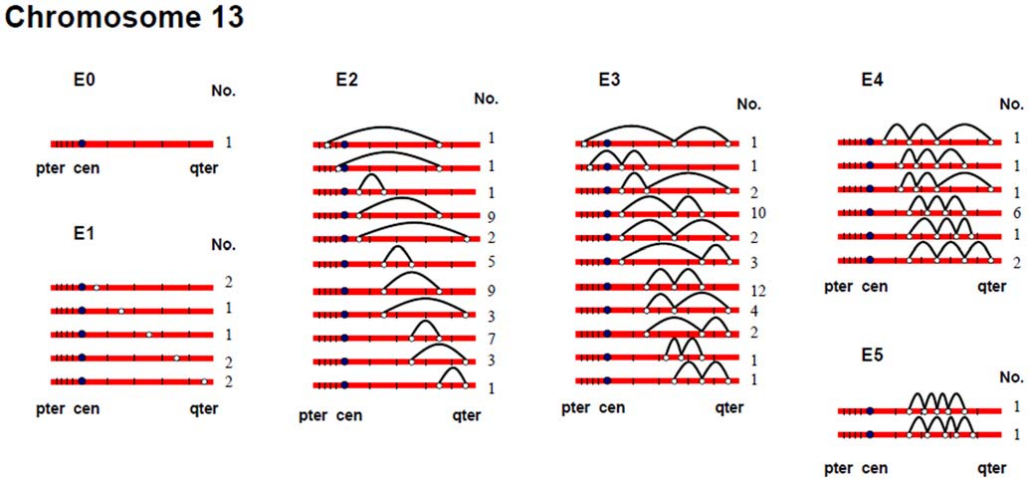
Chromosomal locations of MLH1 foci on chromosome 13 (Figure 2), considered by the number of MLH1 foci per SC.

**Figure 3:**
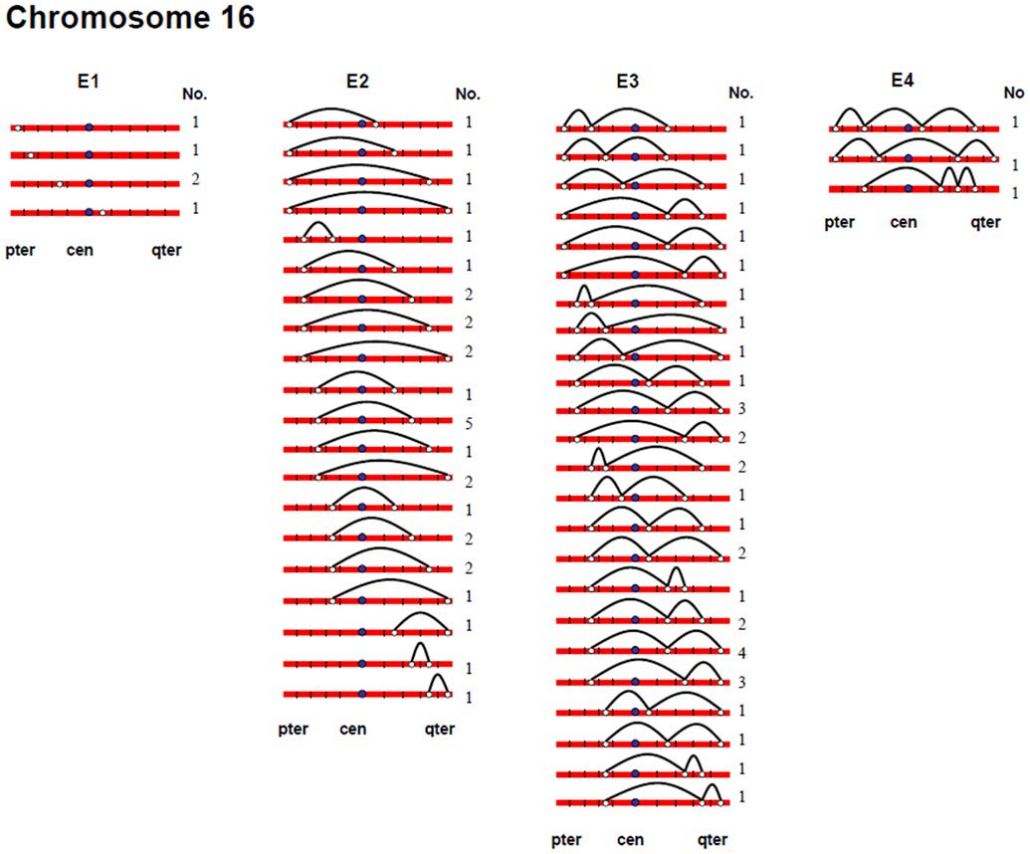
Chromosomal locations of MLH1 foci on chromosome 16, considered by the number of MLH1 foci per SC.

**Figure 4:**
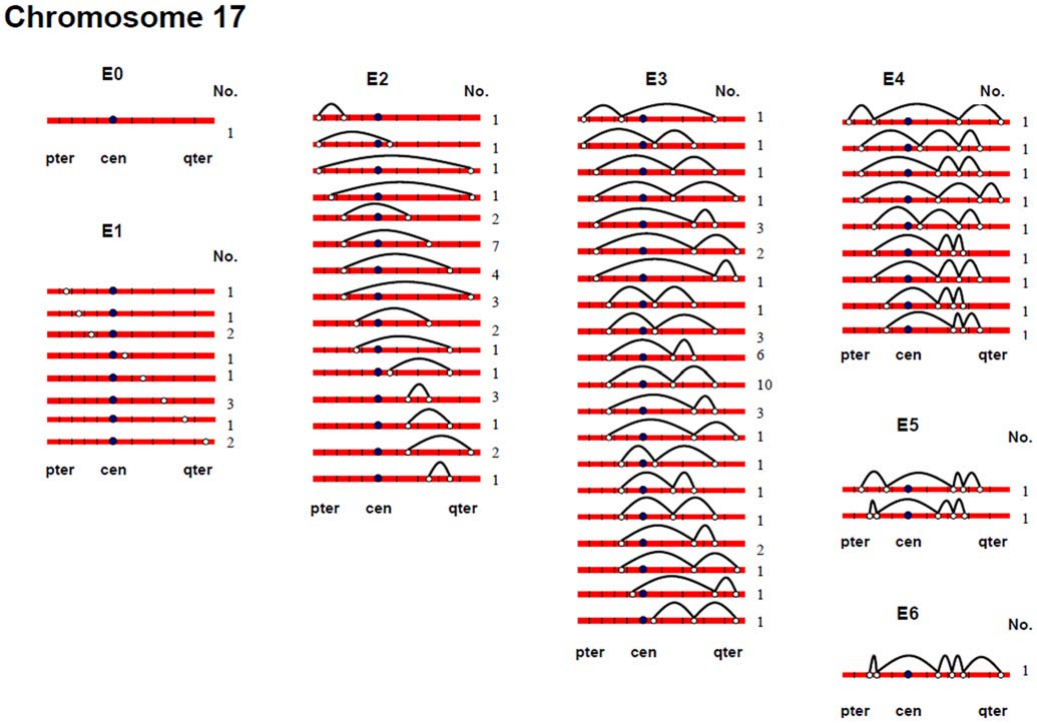
Chromosomal locations of MLH1 foci on chromosome 17, considered by the number of MLH1 foci per SC.

**Figure 5:**
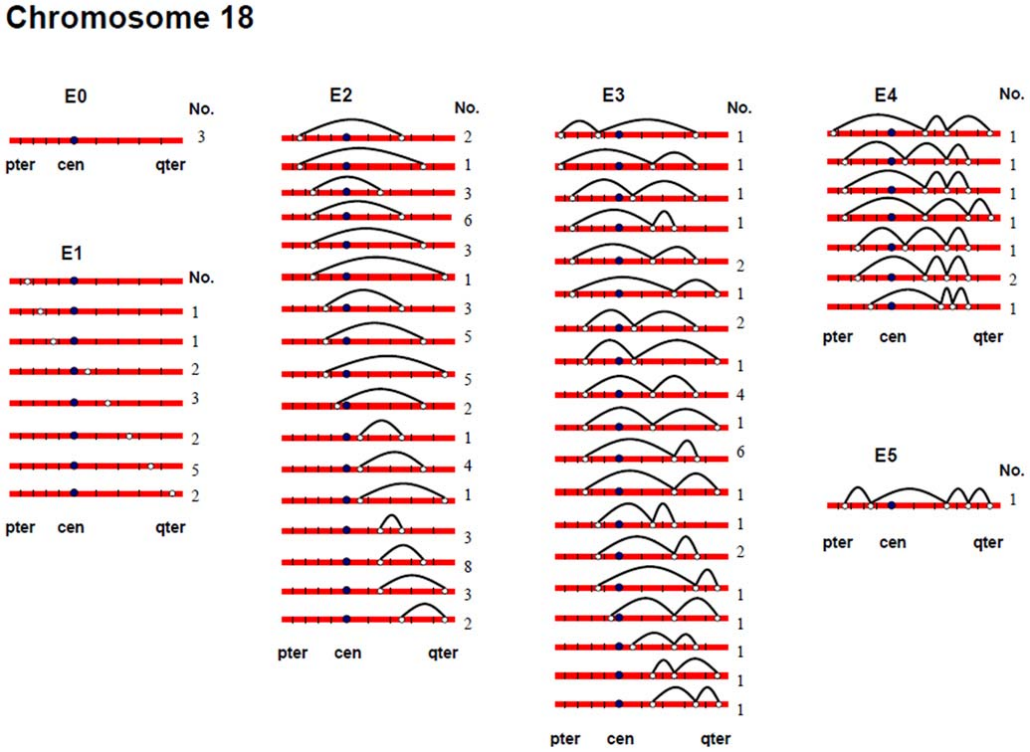
Chromosomal locations of MLH1 foci on chromosome 18, considered by the number of MLH1 foci per SC.

**Figure 6:**
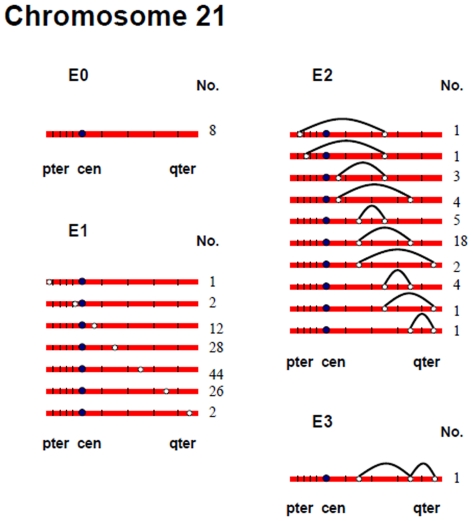
Chromosomal locations of MLH1 foci on chromosome 21, considered by the number of MLH1 foci per SC.

**Figure 7:**
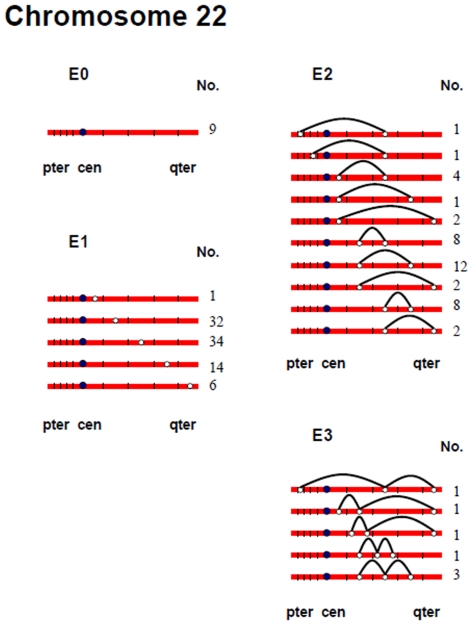
Chromosomal locations of MLH1 foci on chromosome 22, considered by the number of MLH1 foci per SC.

Figure 2, Figure 3, Figure 4, Figure 5, Figure 6, and Figure 7 show the approximate location of MLH1 foci, ordered by the number of MLH1 foci per SC, for chromosomes 13, 16, 17, 18, 21 and 22 (Figure S2, S3, S4, S5, S6, S7 show the same information, but with the data pooled by the number of MLH1 foci per SC). For this analysis, we simply divided the chromosome arms into five equal segments, and specified the segmental location for each MLH1 focus. Clearly, this only provides a general assessment of MLH1 localization, since it does not take into account the variation in arm-arm physical lengths for individual chromosomes. Nevertheless, it provides a mechanism for generating initial cytological maps and for examining spatial relationships between foci that are located on the same chromosomes.

For most chromosome arms, interstitial locations predominated, with the highest proportion of MLH1 foci being medially placed. That is, excluding the short arms of the acrocentric chromosomes, the proportion of foci located in the “middle” segment exceeded the predicted 20% assuming random placement, and typically accounted for between 30% and 60% of foci per arm (Figure S2, S3, S4, S5, S6, S7). In contrast, telomeric and centromeric foci were under-represented on almost all chromosome arms, with each region typically accounting for 10% or fewer of all foci. Chromosome 16 provided the one notable exception to this trend. Specifically, on 16q the highest proportion of MLH1 foci (29%) was located in the most telomeric region; further, the proportion of telomeric foci on 16p (16%) was only slightly less than the 20% expectation and was higher than that observed for any other chromosome arm except 16q.

**Figure 8:**
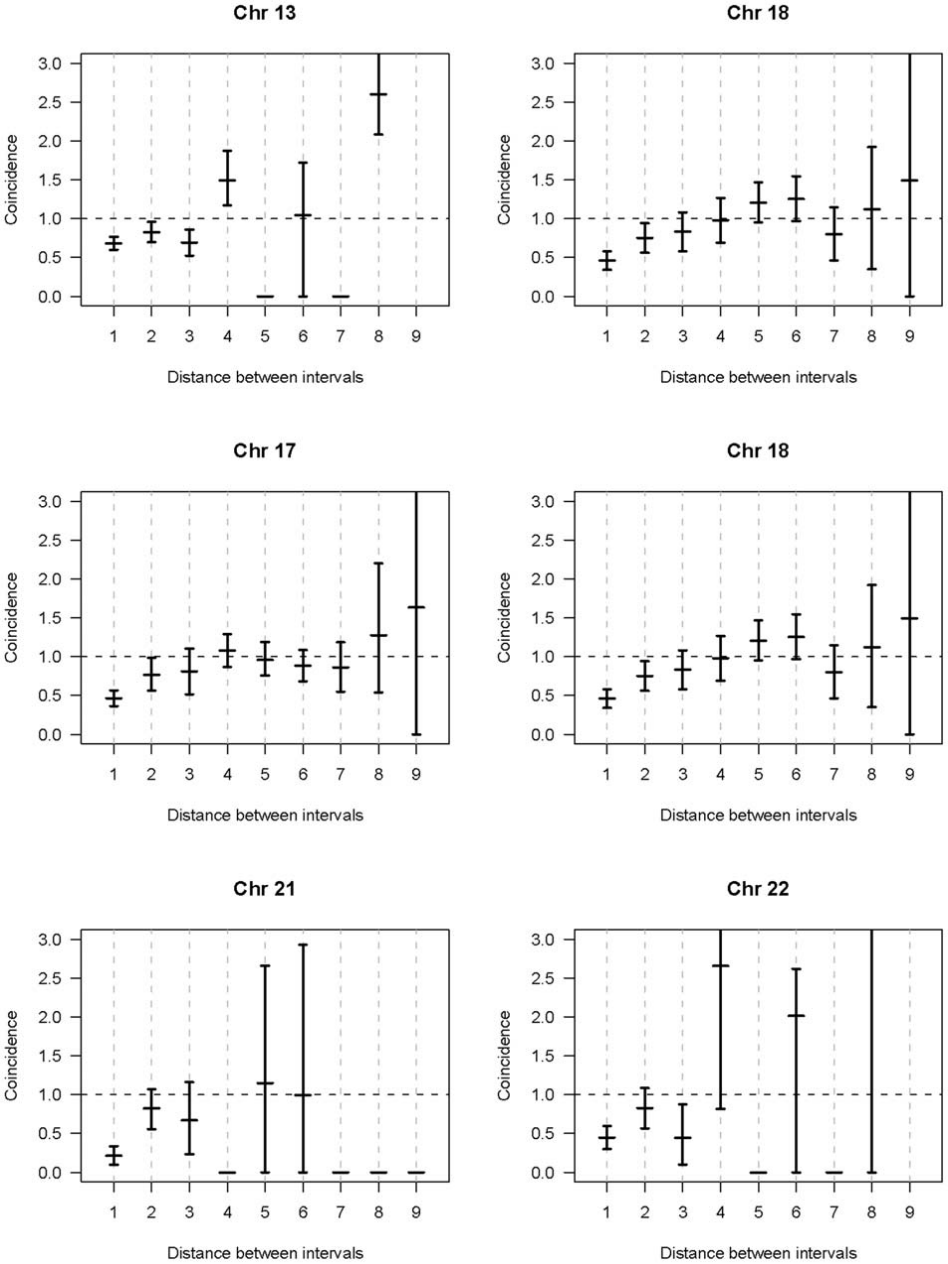
Estimates of coincidence (and 95% confidence intervals) for intervals of different lengths on chromosomes 13, 16, 17, 18, 21, and 22. For this analysis, coincidence was defined as: Pr (MLH1 foci in both intervals)/Pr (MLH1 focus in interval 1)×Pr (MLH1 focus in interval 2). Evidence for positive interference is denoted by coincidence values<1.0. For example, for chromosome 13, coincidence values were significantly under 1.0 over one, two, and three intervals, indicating that the presence of an MLH1 focus inhibited the presence of a second focus over as many as three intervals (e.g., from the p arm telomeric interval to the p arm proximal interval). Similarly, for chromosome 18 the effect extended over two intervals (e.g., from the p arm telomeric interval to the p arm medial interval).

In a separate analysis, we examined the spacing of MLH1 foci on bivalents with two or more foci. For each of the six chromosomes, the placement of foci was clearly non-random, consistent with strong positive interference (Figure 2, Figure 3, Figure 4, Figure 5, Figure 6, Figure 7). For example, in the simplest situation – SCs with exactly two MLH1 foci – the two foci were never observed in the same region (i.e., 0/238 SCs involving chromosomes 13, 16, 17, 18, 21 or 22). Subsequently, we examined interference more directly by estimating coincidence values over different interval distances for each of the chromosomes (Figure 8). When all SCs with multiple foci were considered, estimates of coincidence indicated significant positive interference over one to three chromosome regions. The relative distance over which interference extended varied among the different chromosomes; i.e. estimates of coincidence significantly <1.0 were observed over three adjacent intervals for chromosome 13, over two intervals for chromosomes 16, 17 and 18, and over one interval for chromosomes 21 and 22 (Figure 8).

**Figure 9:**
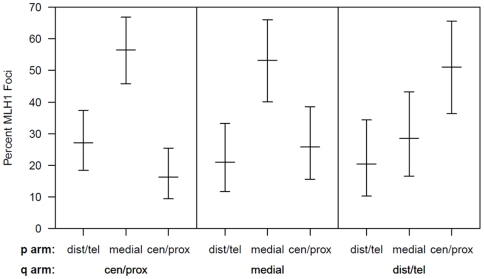
Analysis of the location of adjacent short (p) arm and long (q) arm exchanges on chromosomes 16, 17, and 18. For bivalents involving chromosomes 16, 17, and 18 in which we observed a single MLH1 focus on the p arm and a single MLH1 focus on the q arm, we examined the location of the p arm focus (centomeric/proximal, medial or distal/telomeric), ordered by the location of the q arm focus.

We were also interested in determining whether interference acted across the centromere, or whether the positioning of MLH1 foci on one chromosome arm was independent of focus location on the other arm. For this analysis, we examined the three non-acrocentric chromosomes (i.e., 16, 17 and 18), analyzing all situations in which adjacent MLH1 foci were located on opposite chromosome arms. Specifically, we analyzed the distribution of MLH1 foci on the short arm by the location of the adjacent long arm focus; for both arms, we collapsed the five segments (centromeric, proximal, medial, distal, telomeric) into three because of the relatively small number of centromeric and telomeric MLH1 foci (Figure 9). The short arm localization patterns were highly significantly different for the three categories (χ2 = 20.4; p<0.001), with distal/telomeric short arm foci more likely to be observed in association with centromeric/proximal long arm foci than with distal/telomeric long arm foci. Thus, we conclude that interference operates over the centromere.

## Discussion

The purpose of the present study was threefold: 1) to determine whether analysis of MLH1 foci in pachytene oocytes could provide a reliable approach to studying human female recombination and if so; 2), to use this approach to characterize basic features of female recombination and; 3), to determine whether “vulnerable” chiasma configurations predicted by the two hit model of human nondisjunction [28] are evident in pachytene oocytes.

### Are MLH1 foci reliable markers of crossovers in human ooocytes?

Over the past several years, analyses of pachytene stage meiocytes from human males, mouse males and mouse females have demonstrated a remarkable correlation between the number and location of MLH1 foci and the predicted occurrence of meiotic recombination events (e.g., [21],[23]). Thus, a number of laboratories, including our own, have assumed that MLH1 foci mark the sites of crossovers. However, the evidence that MLH1 localizes to sites of recombination in human female oocytes is not as convincing. For example, both Lenzi et al [17] and Hulten and colleagues [26] have reported lower average MLH1 counts per oocyte than those predicted from genetic linkage analyses of human females (Table 5).

**Table 5:** Estimates of genome-wide levels of recombination in human females.

Method of analysis	No. meioses	Mean no. MLH1 foci	Genetic length (cM)	Reference
MLH1 foci:				
	3	95.0±12.3	4750	19
	95	70.3±10.5	3515	26
	c. 250	50.3±24.7	2515	16
	1035	69.3±14.3	3465	present study
Genetic linkage:	-----	-----	3799	28
	-----	-----	4435	39
	-----	-----	4460	30
	-----	-----	4414	31
	-----	-----	4600	32
	-----	-----	4320	33

The results of the present study are in good agreement with these previous immunostaining studies. In our series the overall mean number of MLH1 foci per oocyte was 69.3, corresponding to a genome-wide genetic length of 3465 cM. As estimates from linkage analyses indicate a female genome-wide length of approximately 4300–4600 cm [29],[30],[31],[32],[33],[34] it means that – similar to the previous immunostaining studies – we may have “missed” approximately 20% of all recombination events.

The reason for this discrepancy is not clear; however, we can think of at least four possible explanations why MLH1-based studies may have underestimated the “real” number of exchanges. First, it could be that these studies selected a sub-set of cells that are unrepresentative of all pachytene stage oocytes. For example, in the present analysis, we excluded cells with obvious synaptic defects. However, since such cells typically contain fewer, not more, MLH1 foci (data not shown), this seems an unlikely explanation. Second, it could be that the discrepancy is attributable to differential selection against a sub-set of oocytes. For example, it may be that, in the human female, the prenatal wave of oocyte atresia is more likely to involve oocytes with low numbers of exchanges so that, on average, oocytes that survive to be ovulated have more exchanges than pachytene stage human fetal oocytes. However, this does not explain the fact that oocyte atresia in female mice does not produce obvious differences in results between MLH1-based assays of recombination and genetic linkage analyses [25],[35]. Third, the discrepancy may reflect the existence of other, non-MLH1-associated crossover pathways. Indeed, non-interfering crossover pathways that are independent of MLH1 have been described in multiple organisms (e.g., [36],[37]), and appear to exist in mammals as well [38]. However, they seem unlikely to play a major role in crossover formation. Cytological [39] and molecular studies [40],[41] of mice homozygous for mutations in MLH1 (or its partner MLH3) suggest that only a small number (10% or less) of crossovers occur in the absence of the MLH1-associated pathway. Thus, while other non MLH1-driven crossover pathways almost certainly contribute to the discrepancy, they provide at best only a partial explanation. Finally, the discrepancy may reflect biological differences in the recombination pathway between human females and human males and mice. In our experience, MLH1 foci are rarely visualized before pachytene in human and mouse males, and are also infrequent in mouse females. In contrast, we and others [17],[26] have observed abundant MLH1 localization in both zygotene and pachytene stage human oocytes, suggesting that crossing-over occurs over a wider temporal window in the human female. If so, crossovers may not be established synchronously in human oocytes, meaning that it will be difficult to visualize all MLH1 foci at the same time. Thus, MLH1 foci would mark exchanges in human oocytes as in human males and mice but, at any given time, not all exchanges would be identifiable.

We favor this last explanation, since it is the only one that fits our observations. Nevertheless, the suggestion of a fundamental difference in the chronology of recombination between human males and females – and between human females and mice – clearly requires confirmation. Initially, it will be important to assess the localization patterns of other recombination proteins in human males and females, and ask whether there are other consistent sex-specific differences.

Regardless of the correctness of this or any of the other possible explanations, one conclusion seems clear – some proportion of exchanges are unrepresented by the MLH1 methodology. Thus it is important that we judiciously interpret MLH1-based data on human female recombination. For example, MLH1 analysis presumably over-estimates the number of achiasmate bivalents, one of the “vulnerable” chiasma configurations associated with models of human nondisjunction. Further, inferences about interference will be complicated by the fact that some exchanges are missed, leading to erroneous conclusions about the chromosomal locations of exchanges. We do not take this to mean that the MLH1-based approach is without merit. Indeed, by comparison with other approaches (e.g., genetic linkage analysis, diakinesis studies of chiasmata), it may provide the most straightforward approach to capturing the vast majority of exchanges in human females. Nevertheless, it does not provide the apparent 1∶1 correspondence that has been observed between MLH1 foci and crossovers in human males and mice.

### What do MLH1 studies tell us about meiotic recombination patterns in human females?

Despite concerns about the utility of the MLH1 approach, the number and distribution of MLH1 foci in pachytene oocytes conformed to two basic principles of meiosis common to most species: first, the presence of at least one exchange per bivalent and second, the non-random positioning of exchanges on chromosomes.

Most importantly, in our analyses virtually all chromosomes contained at least one MLH1 focus. In the 176 cells in which we were able to analyze the number of MLH1 foci on all 23 bivalents, we identified only 57 MLH1-less bivalents. Considered on a per chromosome basis, this means that only 1.4% (57/4048) of all bivalents lacked an MLH1 focus. Subsequent analyses of individual chromosomes 13, 16, 17, 18, 21 and 22 were consistent with the genome-wide observations. Specifically, the proportion of MLH1-less bivalents ranged from a low of 0% for chromosome 16 to highs of approximately 5–6% for chromosomes 21 and 22 (Table 3). While we did not systematically study individual chromosomes 1–12 or the X chromosome, limited analyses of these chromosomes provided little evidence of MLH1-less bivalents (data not shown); thus in our series the values for chromosomes 21 and 22 are undoubtedly the highest for any chromosomes.

Our observations also provide strong evidence for a non-random distribution of exchanges on chromosomes. First, the number of MLH1 foci per chromosome was constrained. For example, for the smallest chromosomes (i.e., 21 and 22), virtually all bivalents had either one or two MLH1 foci, while for chromosomes 13, 16, 17 and 18 the vast majority had two, three or four foci. Second, for each of the six individual chromosomes analyzed, the foci were spread out along the chromosomes; i.e., their placement was consistent with positive crossover interference (Figure 8). Consistent with immunostaining studies of human males [42] and with previous linkage analyses [43], we found no evidence that interference was impeded by the centromere.

The number and distribution of MLH1 foci also recapitulated several observations from human genetic linkage analyses and cytological analyses of recombination. For example, despite our conclusion that we missed some exchanges, the mean number of MLH1 foci per oocyte was still far in excess of that reported for human pachytene spermatocytes (i.e., approximately 50 per cell; [22]). Thus, in agreement with evidence from genetic linkage studies (e.g., [44]), our analyses indicate that meiotic recombination events are more frequent in human females than in human males. Additionally, our observations were consistent with available data on sex-specific differences in the placement of exchanges. On nearly all chromosome arms that we examined, interstitially located foci predominated (Figure 2, Figure 3, Figure 4, Figure 5, Figure 6, Figure 7). This finding agrees with data from human genetic linkage analyses and previous immunostaining studies, which indicate that distal chromosome regions are enriched for recombination in males by comparison with females (e.g., [16]). Finally, consistent with previous MLH1-based data [16],we observed significant among-individual variation in exchange frequencies, with mean MLH1 counts per cell ranging from approximately 60 to 90. Similarly, previous linkage analyses of genome-wide recombination levels in human females have also identified significant individual to individual variation [30], albeit not as pronounced as the differences that we observed.

Thus, in general, our observations on MLH1 foci reinforce several previously reported features of human female recombination. However, there was one surprising difference between our observations and previously reported genetic linkage data. Specifically, our observation of low levels of MLH1-less bivalents is in sharp contrast to previous chromosome-specific estimates of exchangeless chromosomes from linkage analyses. For example, Bugge et al [6] suggested that approximately 12% of chromosome 13 bivalents are achiasmate, while Oliver et al [15] recently reported a value of 20% for chromosome 21. In contrast, in our analysis only 1% of chromosome 13 bivalents and 5% of chromosome 21 bivalents were lacking an MLH1 focus (Table 3). Further, as discussed above, these values likely overestimate the real frequency of MLH1-less bivalents, making the discrepancy between the linkage and MLH1-based data even more puzzling. While it is not possible to know with certainty which of these estimates is “right”, it is instructive to ask which – if either – data set yields the better fit to available data on trisomies from human pregnancies. Assuming random segregation of achiasmate bivalents, our MLH1 data suggest maternal nondisjunction rates of approximately 0.5% for chromosome 13 and 2.5% for chromosomes 21, while the genetic linkage analyses suggest levels of 6% for chromosome 13 [6] and 10% for chromosome 21 [15]. While the actual incidence of trisomies 13 and 21 in human gametes and pregnancies is not known, observations from oocytes, cleavage stage embryos and clinically recognized pregnancies are much closer to the MLH1-based estimates than those implied by the linkage studies. Specifically, cytogenetic studies of oocytes and cleavage stage embryos suggest that trisomy 21 is more common than trisomy 13, but neither condition occurs in more than about 5% of cases (e.g., [45],[46],[47],[48]); and in clinically recognized pregnancies, trisomies 13 and 21 account for only 0.2% and 0.5% of cases, respectively [27]. Taken together, these data suggest that, even in oocytes or very early stage embryos, the observed levels of trisomies 13 and 21 are one-half or less that expected on the basis of the linkage data. Thus, unless humans possess Drosophila-like mechanisms to segregate achiasmate chromosomes (e.g., [49]), it seems likely that MLH1-based assays are better predictors of achiasmate levels than are linkage analyses.

Regardless of the correctness of this conclusion, the discrepant observations beg another question – why do the two methods differ in the first place? Specifically, why does a method that does not capture all crossovers (MLH1 analysis) yield a lower estimate of exchangeless bivalents than a method (linkage analysis) that yields a higher overall level of genome-wide recombination? We can think of a number of reasons why there might be differences between the two approaches, but none of them adequately explains available data on recombination and/or levels of human trisomies. For example, it may be that there is significant recombination-associated selection against a sub-set of oocytes, so that these are eliminated and never contribute to pregnancies (and consequently, are not represented in linkage data sets). However, to reconcile the linkage and MLH1 data the selection would have to favor oocytes with exchangeless bivalents, a phenomenon that – at least on the surface – seems implausible. Further, this has the effect of increasing the expected incidence of trisomies in humans to levels that are simply unrealistic in the absence of an achiasmate segregation mechanism; e.g., to over 5% for trisomies 13 and 21. Second, because we selected the “best” oocytes (those with complete synapsis) for our studies, we may have excluded oocytes with exchangeless bivalents. If these are able to complete meiosis and are capable of being fertilized, the number of zero exchange events might be much higher than we have estimated (e.g., possibly as high as that associated with linkage analysis). However, if this were the case, we are still left with the discrepancy between the observed and expected incidence of trisomies; i.e., the high estimates associated with linkage studies. Third, it may be that a proportion of MLH1 foci do not give rise to crossovers, meaning that we under-estimated the real number of exchangeless homologs. However, if this were the case, the problems associated with the above explanations still apply; additionally, we are unaware of data from any species suggesting that only some MLH1 foci contribute to crossovers. Finally, it may be that, at least for some chromosomes, linkage analysis systematically underestimates the actual number of exchanges. For example, marker panels might not adequately cover all regions (e.g., extremely distal or proximal segments), resulting in missed exchanges. However, while this would bring the linkage data in line with our observations it would create another problem, since the discrepancy between the MLH1-based and linkage-based estimates of genome-wide recombination estimates would be even greater.

Thus, the reason for the discrepancy between the two approaches is not immediately obvious. Clearly, it is important that future analyses address this issue, since accurate data on the number and chromosome-specific nature of exchangeless bivalents is central to our understanding of human meiosis and mechanisms of meiotic nondisjunction.

### Are there chromosome-specific differences in the frequency of “vulnerable” chiasma configurations?

Alterations in either the number or placement of recombination events have been implicated in the genesis of all human trisomies that have been appropriately studied. In general, these reports suggest there are three types of chiasma configurations that predispose to nondisjunction in humans: bivalents that have no exchanges, bivalents with exchanges too far from the centromere, and bivalents with exchanges too close to the centromere [14]. However, as outlined in Table 6, the nature of these alterations varies for maternally-derived trisomies involving different chromosomes.

**Table 6:** Summary of data correlating recombination defects with the genesis of maternally-derived trisomies.

Trisomy	Achiasmate bivalents	Distal exchanges	Proximal exchanges	Comments (references)
13	yes	no	no	Estimated 25–33% of cases associated with achiasmate bivalents (2, 3)
16	no	yes	no	No known contribution of achiasmate bivalents, but distally located exchanges reported for most cases (5, T. Hassold and H. Hall, unpublished observations)
17	unknown	unknown	unknown	“Rare” trisomy; no available information on origin
18	yes	no	no	Estimated 30% of cases associated with achiasmate bivalents (6)
21	yes	yes	yes	Estimated 40% of cases involve achiasmate bivalents; distally located exchanges important contributor to meiosis I cases and proximal exchanges important to apparent meiosis II cases (7, 8)
22	yes	no	no	Estimated 25% of cases associated with achiasmate bivalents (9)

Presumably, these chromosome-specific differences could originate in one of two ways. First, they could originate prenatally, at the time that crossovers are formed. That is, it may be that the likelihood of specific types of “vulnerable” chiasma configurations varies among chromosomes and that, once established, similar proportions of these are translated into nondisjunctional events. If this is the case, the recombination patterns observed in linkage analyses of individual trisomies should be reflected by the number and chromosomal location of MLH1 foci in pachytene oocytes. For example, for trisomy 18, achiasmate bivalents are an important risk factor but “misplaced” (extremely proximal or distal) exchanges are not; thus, in analyses of pachytene oocytes we might expect to identify a relatively high proportion of MLH1-less chromosome 18 bivalents but few, if any, with extremely proximal or distal MLH1 foci.

Alternatively, it might be that the chromosome-specific differences arise post-recombination in the adult ovary, with the ability to process vulnerable chiasma configurations varying among chromosomes. This might occur if chromosome-specific differences in the distribution of repetitive elements affect the binding of chromosome-associated proteins important for segregation. For example, if the large block of heterochromatin on 16q interferes with alignment of homologous centromeres on the metaphase I plate, proximal exchanges might be more important for segregation of chromosome 16 than for chromosome 17. In this instance, there is no reason to invoke chromosome-specific differences in the types or frequency of vulnerable chiasma configurations in pachytene oocytes. Instead, the chromosome-specific differences arise because of variation in the ability of individual chromosomes to process sub-optimal chiasma configurations.

Which, if either, of these alternatives fits the data from the present study? For most trisomic situations it appears to be the first, since the linkage results on trisomies are reflected by the MLH1 data on pachytene oocytes. For example, in accordance with the linkage data on trisomy 16, we found no evidence for achiasmate chromosomes 16. For chromosome 18, the data from trisomies predict a high proportion of achiasmate bivalents but no unusually placed exchanges. Consistent with this prediction, approximately 3% of bivalents lacked MLH1 foci and in a further 37.5% MLH1 foci were missing on either the long or short arm; further there was little evidence for extremely proximal or distal exchanges. Finally, for chromosomes 21 and 22 we expected a high incidence of achiasmate bivalents and these predictions were met, with MLH1-less bivalents observed in no fewer than 4.9% and 6.3% of cases, respectively.

Not all situations were as clear-cut. For example, we found no evidence for “spikes” in the proportion of extremely distal or proximal MLH1 foci on chromosome 21, despite the fact that proximal and distal exchanges are associated with maternal meiosis I and II-derived cases of trisomy 21 [13],[15]. Additionally, the proportion of MLH1-less chromosomes 13 was negligible, despite the fact that an estimated 25–33% of maternally-derived cases of trisomy 13 are thought to arise from achiasmate meioses [3]. Thus, there was not complete concordance between the MLH1 data on oocytes and the linkage data on trisomies. Nevertheless, taken as a whole, the results provide strong evidence that at least some of the chromosome-specific differences in nondisjunction patterns are established during meiotic prophase and, more generally, that some chromosomes are pre-disposed to nondisjoin because of events that occurred in the fetal ovary. The mechanisms by which these susceptibilities are “translated” into nodisjunctional events years later are not clear, nor is the way in which maternal age acts on the different aberrant exchange configurations. For example, exchangeless bivalents presumably impart a risk of nondisjunction regardless of maternal age, but the effects of pericentromeric and telomeric exchanges on segregation likely vary with age and among the different chromosomes. Clearly, an eventual understanding of human nondisjunction will require us to consider the effects of recombination and maternal age separately for each chromosome, since it is now evident that no one trisomy will serve as a paradigm for all such conditions.

## Materials and Methods

### Ethics statement

This study was conducted according to the principles expressed in the Declaration of Helsinki. All procedures were approved by the University of California-San Francisco, University of Washington and Washington State University Institutional Review Boards, and informed consent was obtained from all study participants.

### Study population

The study material consisted of 1035 prophase oocytes from 31 fetal ovarian samples, with gestational ages ranging between 14–23 weeks, collected at the University of Washington Medical Center in Seattle, Washington, or at the San Francisco General Hospital Women's Options Center in San Francisco, California (Table 1). Typically, fetal ovaries were isolated and processed within 24 hours of the surgical procedure. Karyotypic information was available on four cases, all of which had a 46,XX chromosome complement; none of the other 27 cases were suspected to have a chromosome abnormality.

### Slide preparation and immunostaining

From collection of sample material, tissues were processed using a standard surface-spreading technique [50]. Briefly, the ovaries were isolated and excess connective tissue removed. Each ovary was placed in a sterile watch glass, covered in ∼2 ml of a hypo-extraction buffer (600 mM TRIS, 500 mM sucrose, 170 mM citric acid, 500 mM EDTA, 500 mM DDT and 100 mM PMSF in distilled water) and incubated at room temperature for 45 minutes. Each ovary was cut into two sections and each section separately suspended in ∼75 µl of 100 mM sucrose. After macerating the ovarian tissue with needles, 10 µl aliquots of the cell suspension were spread across glass slides coated with 2% paraformaldehyde (pH 9.2). Slides were kept overnight in a humidified chamber.

Slides were washed in 0.04% Photoflo™ in double-distilled water for 2 minutes and air-dried. The slides were then pre-incubated for 20 minutes at room temperature in 1× antibody dilution buffer (ADB). Sixty µl of an antibody cocktail consisting of MLH1 (1∶75; BD Pharmingen mouse anti-human) and CREST (1∶1,000; Fisher Scientific human anti-centromere) was applied to the slides, which were then incubated overnight at 37°C. Sixty µl of SYCP3 (1∶150; Novus Biologicals rabbit anti-human polyclonal) was added to the slides. The slides were covered with parafilm and incubated for 2 hours at 37°C. Subsequently, the slides were washed twice in 1× ADB for 20 minutes and a 60 µl cocktail consisting of fluorescein anti-mouse (1∶75) and CREST anti-human (1∶100) was added. Slides were incubated overnight at 37°C and 60 µl of rhodamine anti-rabbit (1∶200) was added to the slides. The slides were covered with parafilm and incubated for 45 minutes at 37°C; the slides were then washed twice in PBS. A drop of FluoroGuard Antifade Reagent (BioRad Laboratories) was added to the slides, which were kept at 4°C until viewing under fluorescence optics. Slides were evaluated on a Zeiss epifluorescence microscope, images captured and cell coordinates noted for subsequent fluorescence in situ hybridization (FISH) analysis (see below).

### Fluorescence in situ hybridization (FISH)

Chromosome-specific FISH was performed on slides that contained meiotic cells with robust MLH1 and CREST signals on initial analysis. Specifically, we used TelVysion13-Spectrum Orange, CEP16-SpectrumGreen, TelVysion17-SpectrumGreen, TelVysion18-SpectrumOrange, TelVysion21-SpectrumOrange and TelVysion22-SpectrumOrange (Vysis). Briefly, previously immunostained slides were dehydrated in an ethanol series (75%, 95% and 100%) at room temperature, denatured in 70% formamide/2× SSC at 73°C for 5 minutes and again dehydrated in an ethanol series (75%, 95% and 100%) at room temperature. The probe mix was denatured using the same settings except for the dehydration step. The probe mix was then added to the slides, which were kept in a humidified chamber at 37°C overnight. Slides were briefly washed in 0.4× SSC, 2× SSC/1% NP-40 and distilled water for 10 seconds, 3 seconds and 1 second, respectively, air-dried and stained with Vectashield (Vector Laboratories) and analyzed using fluorescence optics. Cells that had previously been analyzed in immunostaining studies were located, and FISH images captured.

### Cytological analysis

In determining the number and location of MLH1 foci in pachytene oocytes, we restricted our analyses to cells in which synapsis was complete, or nearly so, and to cells with MLH1 signals of robust size, shape, staining intensity and association with the synaptonemal complex (SC). All such cells were scored, with no attempt made to restrict our analysis to cells with an arbitrary minimum number of MLH1 foci. Apparent MLH1 signals that were observed at overlapping locations between two or more SCs were not counted; additionally, to be considered as separate foci, we required that the space between adjacent MLH signals be equal to at least one MLH1 signal domain. All cells were scored by at least two observers; in the event of discrepancies, the cells were omitted.

For analyses of individual chromosomes, we used FISH to analyze approximately 100 cells for six chromosomes (13, 16, 17, 18, 21 and 22) from a subset of seven representative cases (EC 69, 76, 91, 96, 98, 99 and 101). FISH-identified SCs involving chromosomes 13, 16, 17, 18, 21 and 22 were analyzed by counting the number of MLH1 foci per SC and per SC arm (p-arm and q-arm). Each SC arm was arbitrarily divided into five segments of equal length (centromeric, proximal, medial, distal and telomeric) and the number and location of MLH1 foci along the SC recorded.

### Analysis of interference

We define the coincidence for the pair of intervals (*i*, *j*) to be *C_ij_* = *π_ij_*/(*π_i_ π_j_*), where *π_ij_* is the probability of crossovers (MLH1 foci) in both intervals *i* and *j*, and *π_i_* is the probability of a crossover in interval *i*. (There were few instances of multiple crossovers within an interval, and so we will define coincidence using the probabilities of at least one crossover in the respective intervals.) Let *p_i_* and *p_ij_* denote our estimates of *π_i_* and *π_ij_*, respectively (that is, the observed proportions of meioses with crossovers in the interval or interval pair). Note that coincidence = 1 corresponds to independence (no crossover interference), while in the case of positive crossover interference, coincidence will be <1. With the assumption that the coincidence is constant for pairs of intervals that are a given distance apart, we estimate the coincidence to be Σ*p_ij_*/Σ(*p_i_ p_j_*), where the sums are over pairs of intervals that are separated by a fixed distance. This estimate is similar to taking the average of the individual estimates, *p_ij_*/(*p_i_ p_j_*), but the ratio of the sums provides a more stable estimate (that is, one with a smaller standard error); this was confirmed by computer simulations.

Confidence intervals for the coincidence values were derived by a nonparametric bootstrap [51]. That is, we sampled with replacement from the observed set of meioses to obtain a new data set of the same size (with some meioses omitted and some repeated multiple times), estimated the coincidence for all possible distances between intervals, and repeated this process 10,000 times. The interval defined by the 2.5 and 97.5 percentiles of the coincidence estimates across bootstrap replicates provides an approximate 95% confidence interval.

## Supporting Information

Figure S1 Distribution of the number of MLH1 foci/cell for 1,035 pachytene oocytes from 31 fetal ovarian samples.
(4.66 MB TIF)

Figure S2 Chromosomal locations of MLH1 foci on chromosomes 13; data represent pooled observations from seven fetal ovarian samples (EC 69, 76, 91, 96, 98, 99, and 101). For each chromosome, the data are grouped by the number of MLH1 foci per bivalent, and second pooled for all the individual groups; n = the number of cells. For example, for chromosome 13, 8 cells had a single MLH1 focus, 42 cells had two foci, 39 had three foci and 14 cells had four or five foci; in total, we examined the distribution of MLH1 foci on chromosome 13 in 103 cells.
(3.71 MB TIF)

Figure S3 Chromosomal locations of MLH1 foci on chromosome 16; see Figure S2 legend.
(4.04 MB TIF)

Figure S4 Chromosomal locations of MLH1 foci on chromosome 17; see Figure S2 legend.
(1.43 MB TIF)

Figure S5 Chromosomal locations of MLH1 foci on chromosome 18; see Figure S2 legend.
(1.44 MB TIF)

Figure S6 Chromosomal locations of MLH1 foci on chromosome 21; see Figure S2 legend.
(3.04 MB TIF)

Figure S7 Chromosomal locations of MLH1 foci on chromosome 22; see Figure S2 legend.
(3.10 MB TIF)
