# Supplementary figures and images for: Meiotic Recombination in Human Oocytes
Source: PLoS Genet. 2009 Sep 18;5(9):e1000661. doi: 10.1371/journal.pgen.1000661 (PMC2735652; doi:10.1371/journal.pgen.1000661)

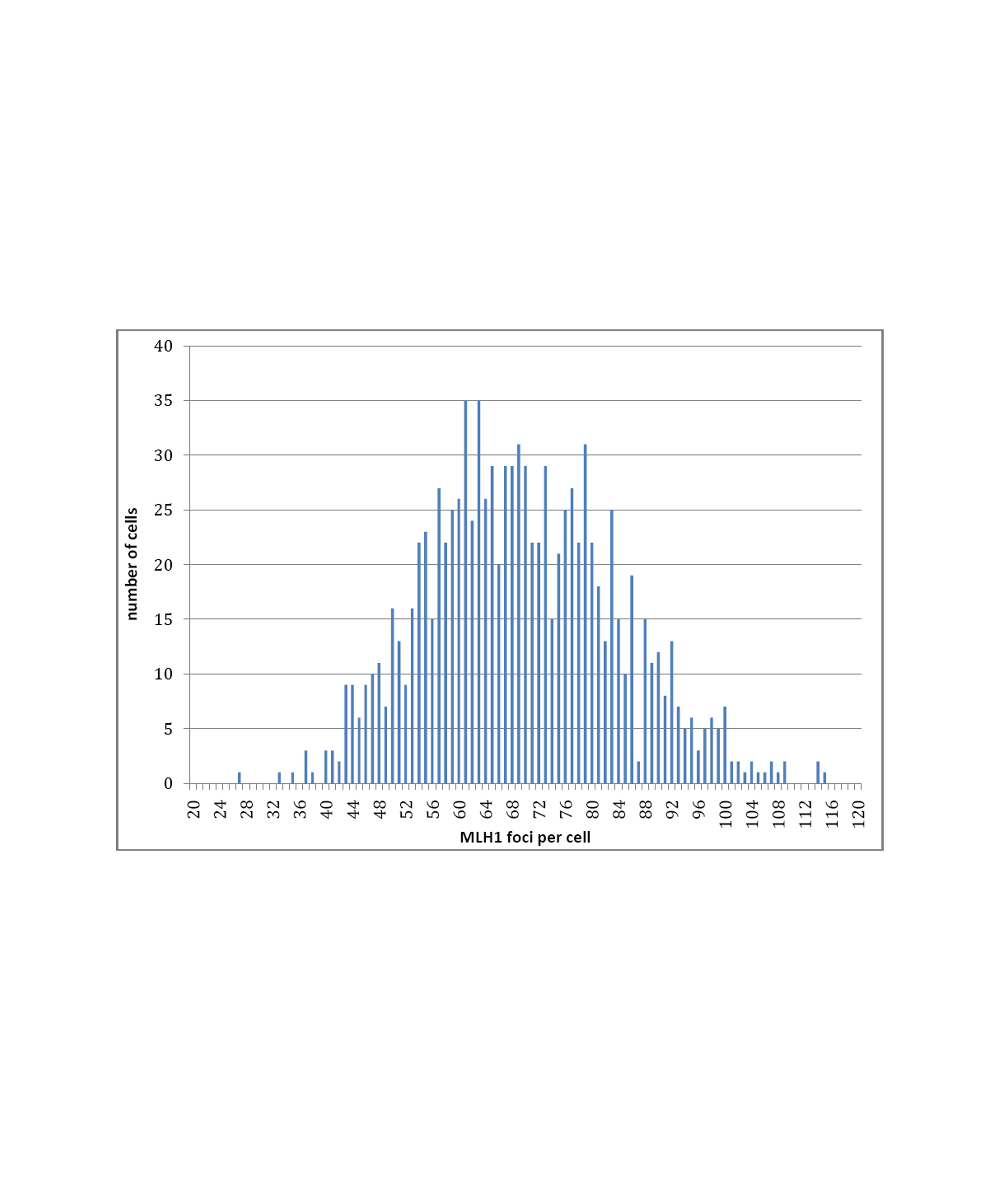

Supplement: Figure S1 — Distribution of the number of MLH1 foci/cell for 1,035 pachytene oocytes from 31 fetal ovarian samples. (4.66 MB TIF) [file pgen.1000661.s001.tif]

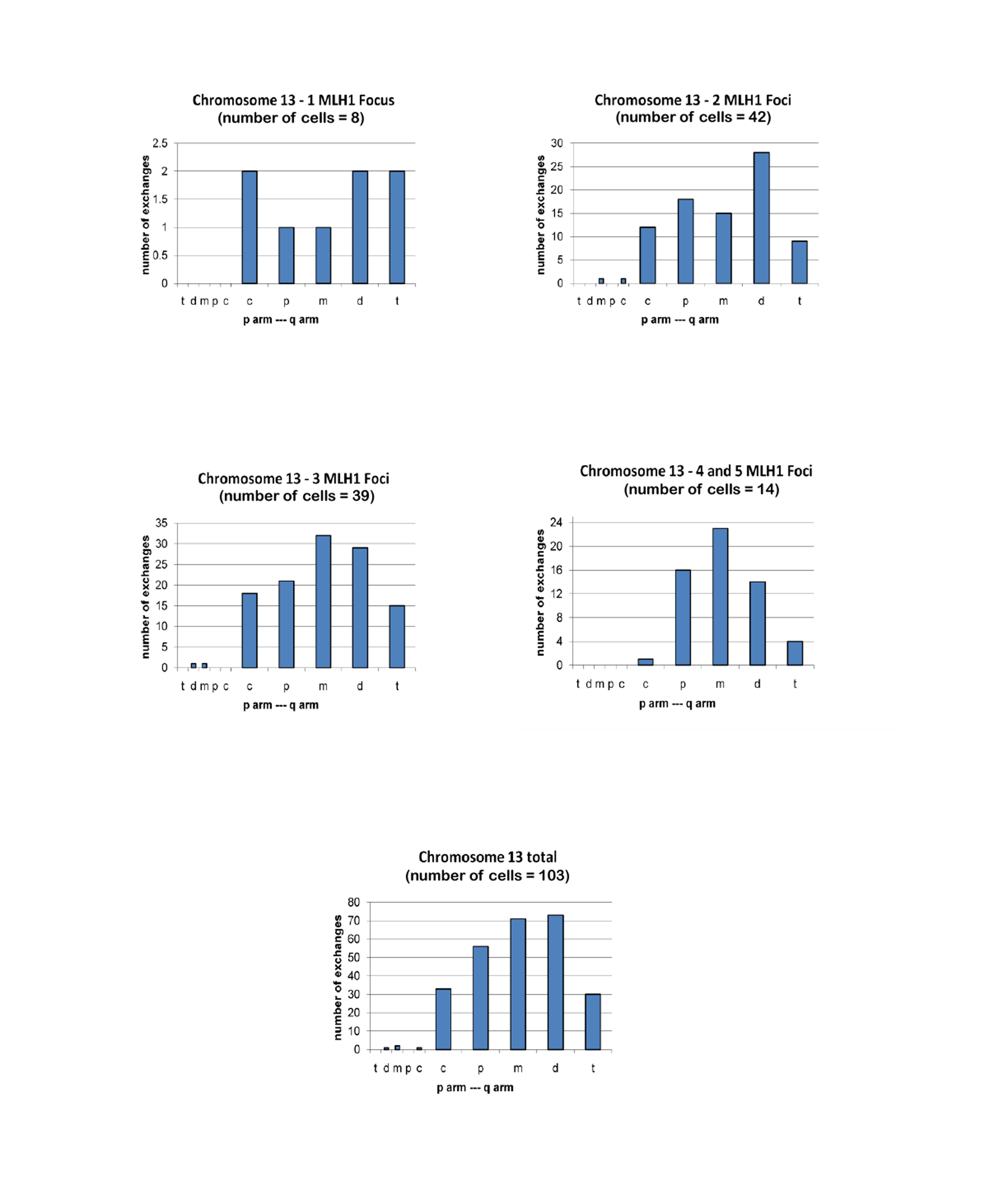

Supplement: Figure S2 — Chromosomal locations of MLH1 foci on chromosomes 13; data represent pooled observations from seven fetal ovarian samples (EC 69, 76, 91, 96, 98, 99, and 101). For each chromosome, the data are grouped by the number of MLH1 foci per bivalent, and second pooled for all the individual groups; n = the number of cells. For example, for chromosome 13, 8 cells had a single MLH1 focus, 42 cells had two foci, 39 had three foci and 14 cells had four or five foci; in total, we examined the distribution of MLH1 foci on chromosome 13 in 103 cells. (3.71 MB TIF) [file pgen.1000661.s002.tif]

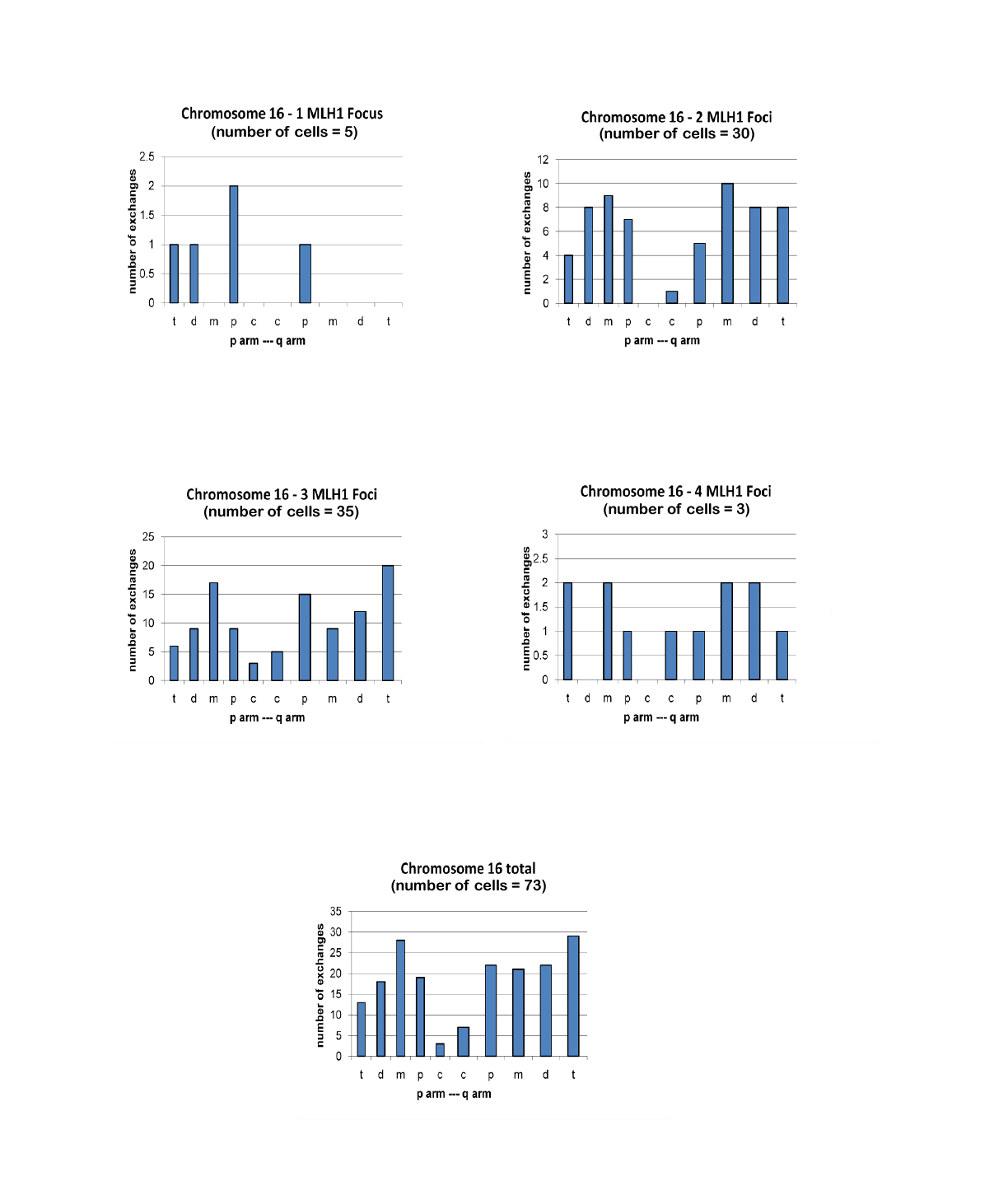

Supplement: Figure S3 — Chromosomal locations of MLH1 foci on chromosome 16; see Figure S2 legend. (4.04 MB TIF) [file pgen.1000661.s003.tif]

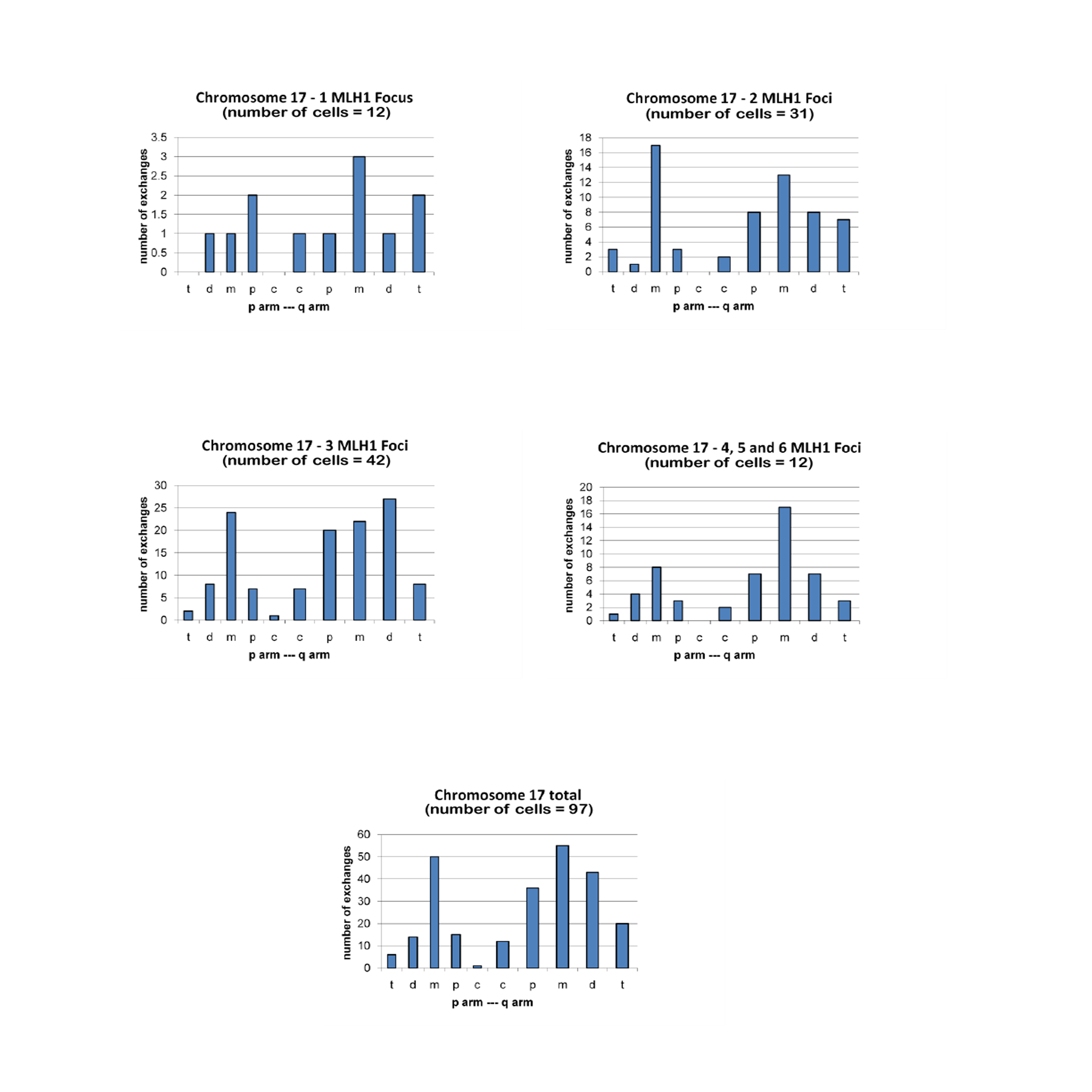

Supplement: Figure S4 — Chromosomal locations of MLH1 foci on chromosome 17; see Figure S2 legend. (1.43 MB TIF) [file pgen.1000661.s004.tif]

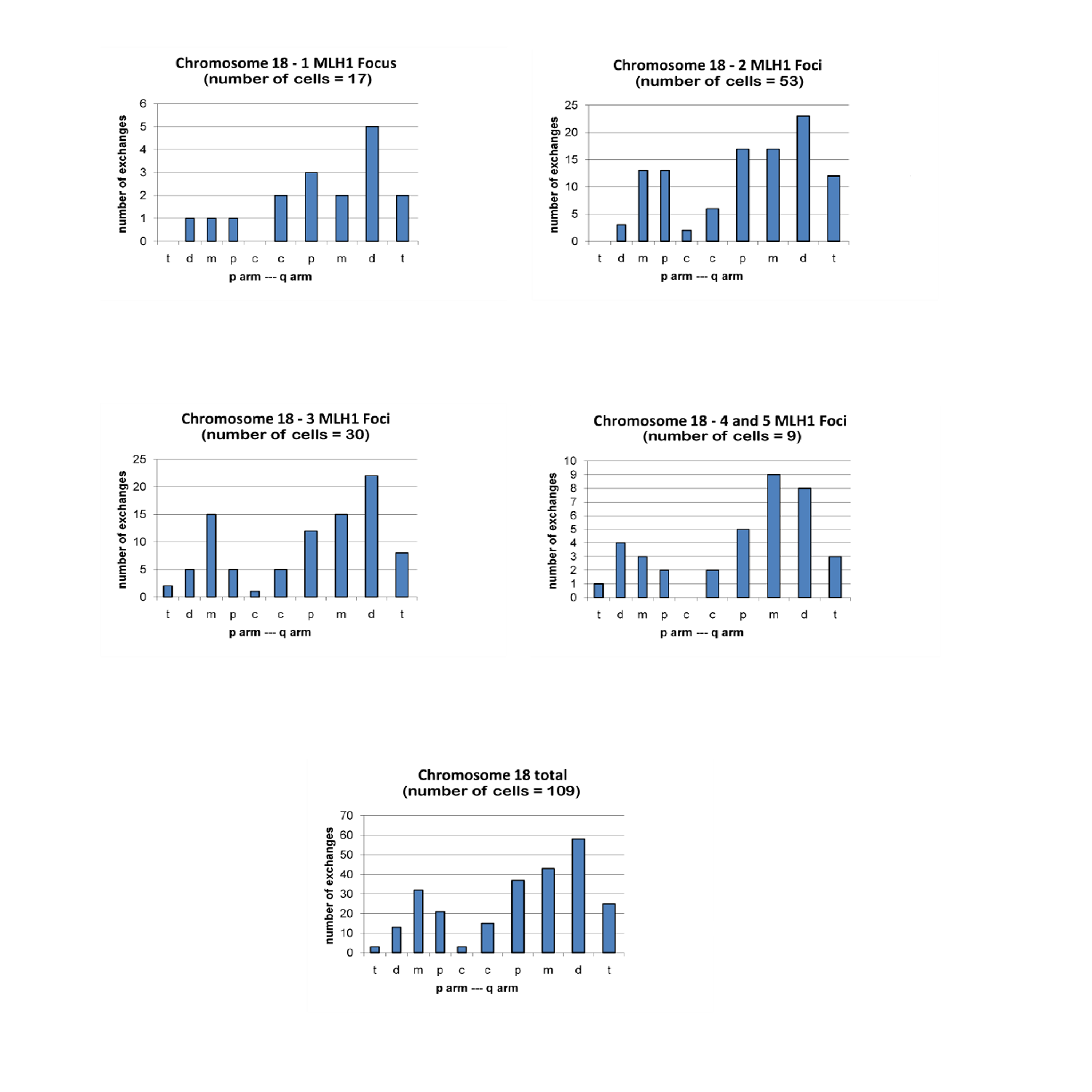

Supplement: Figure S5 — Chromosomal locations of MLH1 foci on chromosome 18; see Figure S2 legend. (1.44 MB TIF) [file pgen.1000661.s005.tif]

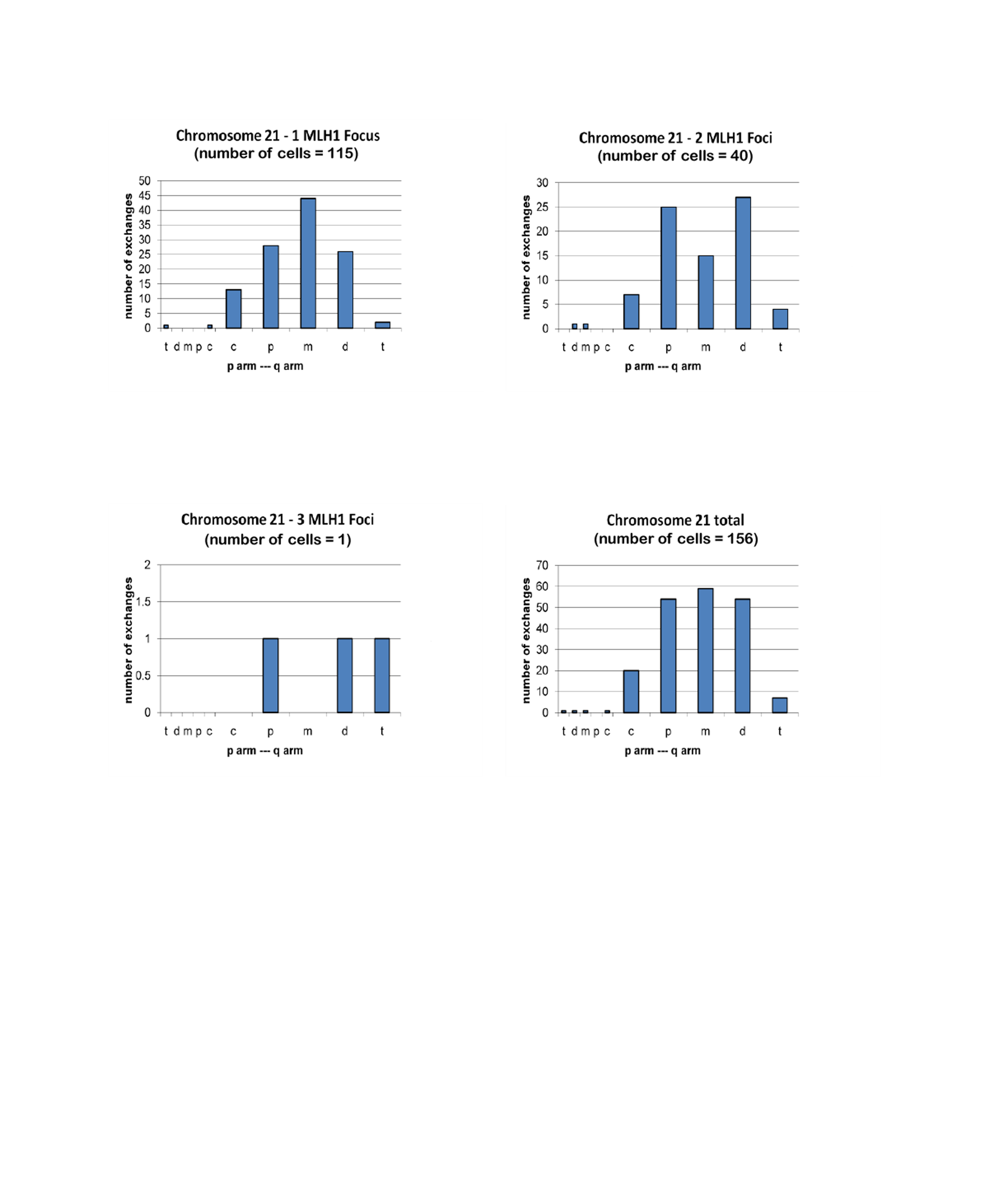

Supplement: Figure S6 — Chromosomal locations of MLH1 foci on chromosome 21; see Figure S2 legend. (3.04 MB TIF) [file pgen.1000661.s006.tif]

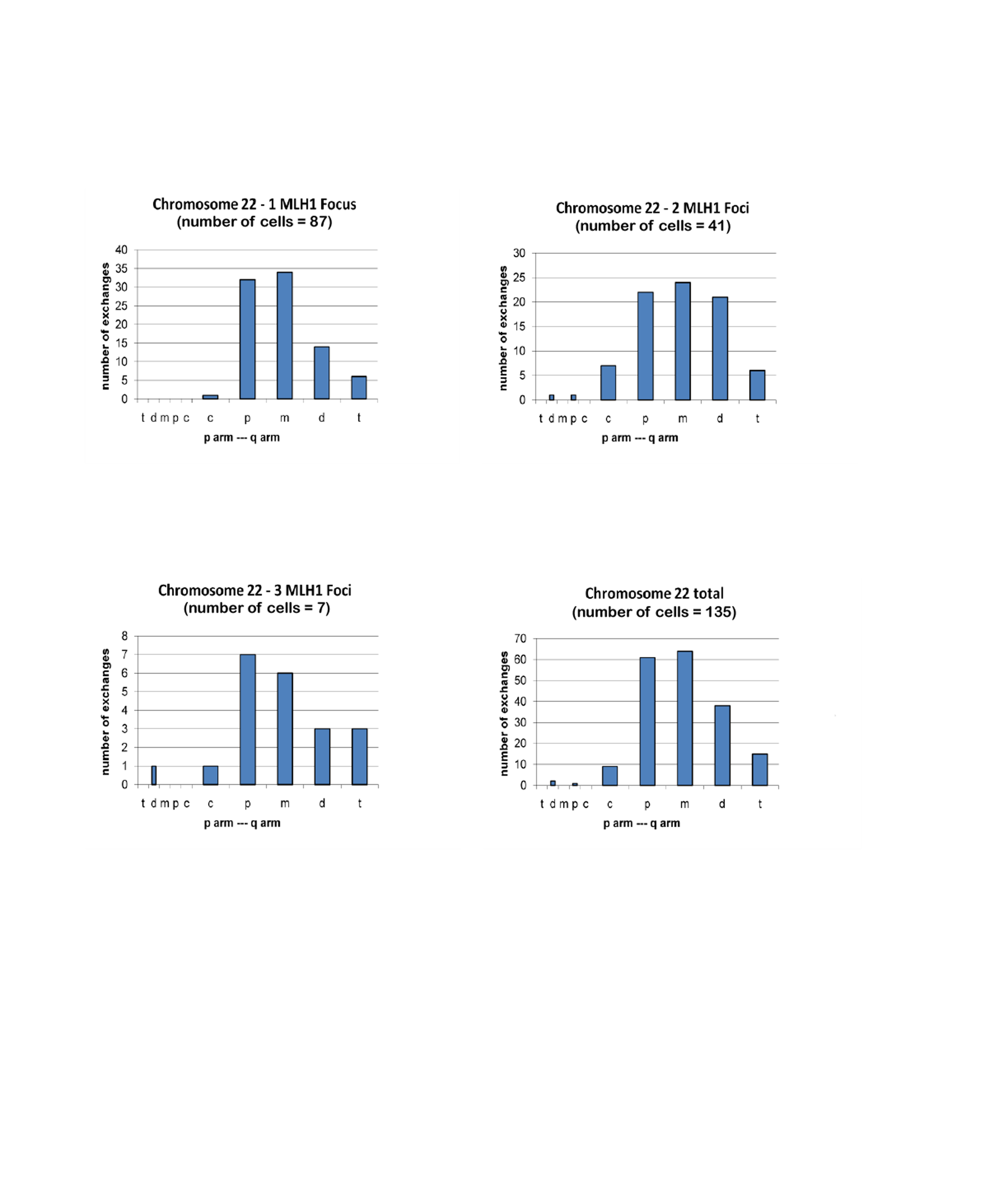

Supplement: Figure S7 — Chromosomal locations of MLH1 foci on chromosome 22; see Figure S2 legend. (3.10 MB TIF) [file pgen.1000661.s007.tif]
